# Case Report: Chronic Prostatitis as an Extraintestinal Manifestation of Ulcerative Colitis

**DOI:** 10.1002/iju5.70083

**Published:** 2025-08-15

**Authors:** Naoya Sugihara, Ryuta Watanabe, Noriyoshi Miura, Takashi Saika, Katsuhisa Ohashi

**Affiliations:** ^1^ Department of Urology Ehime University School of Medicine Toon‐shi Japan; ^2^ Ohashi Gastroenterology and Proctologic Surgery Clinic Niihama Japan

**Keywords:** chronic prostatitis, extraintestinal manifestations, inflammatory bowel disease, lower urinary tract symptoms, ulcerative colitis

## Abstract

**Introduction:**

Lower urinary tract symptoms are common in patients with inflammatory bowel disease; however, the association between ulcerative colitis and chronic prostatitis remains underrecognized.

**Case Presentation:**

A 38‐year‐old man presented with frequent and painful urination unresponsive to the standard treatment of chronic prostatitis. He was subsequently diagnosed with ulcerative colitis based on persistent hematochezia and colonoscopy findings. Treatment with mesalamine and corticosteroids for ulcerative colitis led to improvements in both gastrointestinal and urinary symptoms.

**Conclusion:**

This case suggests a possible link between chronic prostatitis and ulcerative colitis, indicating that prostatitis might represent an extraintestinal manifestation. This association might be explained by shared inflammatory pathways and the prostate's anatomical proximity to the rectum. Clinicians should consider inflammatory bowel disease in male patients exhibiting refractory chronic prostatitis and concurrent gastrointestinal symptoms.

**Abbreviations**
EIMs: extraintestinal manifestations
IBD: inflammatory bowel disease
IBS: irritable bowel syndrome
IL: interleukin
LUTS: lower urinary tract symptoms
NIH: National Institutes of Health
NIH‐CPSI: NIH‐chronic prostatitis symptom index
PSA: prostate‐specific antigen
QoL: quality of life
TNF: tumor necrosis factor
UC: ulcerative colitis

**Summary**

- This case highlights chronic prostatitis as a potential extraintestinal manifestation of ulcerative colitis.
- In a patient with treatment‐resistant urinary symptoms, addressing the underlying intestinal inflammation led to significant improvements in both gastrointestinal and urological symptoms.
- This suggests that inflammation‐driven conditions, such as ulcerative colitis might contribute to chronic prostatitis through shared immune pathways.
- Recognizing this link could help guide more accurate diagnoses and effective treatment strategies.

## 1 Introduction

Ulcerative colitis (UC) is a chronic inflammatory bowel disease (IBD) limited to the colonic mucosa and known for its extraintestinal manifestations (EIMs) involving the skin, joints, eyes, and hepatobiliary system [1]. Recent studies have also reported a high prevalence of lower urinary tract symptoms (LUTS) in patients with IBD [2]. However, these associations have not been adequately characterized in urologic literature.

Chronic prostatitis, which affects approximately 2%–10% of men, manifests persistent LUTS and pelvic discomfort; nonetheless, its etiology remains multifactorial and poorly understood [3, 4]. Although the co‐occurrence of UC and chronic prostatitis has rarely been reported, emerging evidence suggests shared inflammatory and immunological mechanisms.

Herein, we present a case of concurrent chronic prostatitis and UC, wherein UC treatment improved prostatic symptoms; supporting a potential pathogenic link between the two conditions.

## 2 Case Presentation

A 38‐year‐old man without significant medical history presented with dysuria and daytime urinary frequency occurring approximately every 30 min for several months. He also reported six–seven bowel movements per day. Initial urinalysis showed unremarkable results. Urine cytology revealed no atypical cells. Prostate‐specific antigen (PSA) levels were 1.10 ng/mL. Ultrasonography revealed only prostatic calcification, with a prostate volume of 20 mL.

**FIGURE 1:**
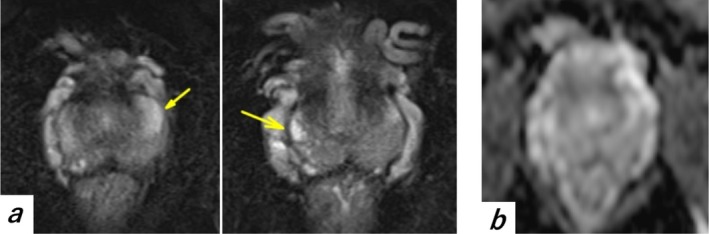
Pelvic magnetic resonance imaging (MRI) of the prostate. (a) T2‐weighted image showing high‐intensity signals (arrows) in the peripheral zone of both lobes, suggestive of inflammation. (b) Apparent diffusion coefficient (ADC) map showing no diffusion restriction in the same region, which is inconsistent with malignancy.

Treatment with naftopidil, levofloxacin, and fesoterodine did not improve the symptoms. Pelvic magnetic resonance imaging revealed hyperintense signals in the prostate peripheral zone on T2‐weighted images (Figure 1), suggestive of inflammation. Although prostatic massage was performed to obtain expressed prostatic secretions (EPS), no fluid could be collected. A post‐massage urine sample (VB3) was obtained; however, it showed no evidence of pyuria or bacterial growth. A diagnosis of National Institutes of Health (NIH) Category IIIb chronic prostatitis was made based on an initial NIH‐chronic prostatitis symptom index (NIH‐CPSI) score of 20 (pain, 6; voiding, 5; Quality of Life (QoL), 9). Symptom evaluation according to the UPOINTS system indicated involvement of the urinary and organ‐specific domains: urinary, psychosocial, organ‐specific, infectious, neurological/systemic, and tenderness of skeletal muscles.

**FIGURE 2:**
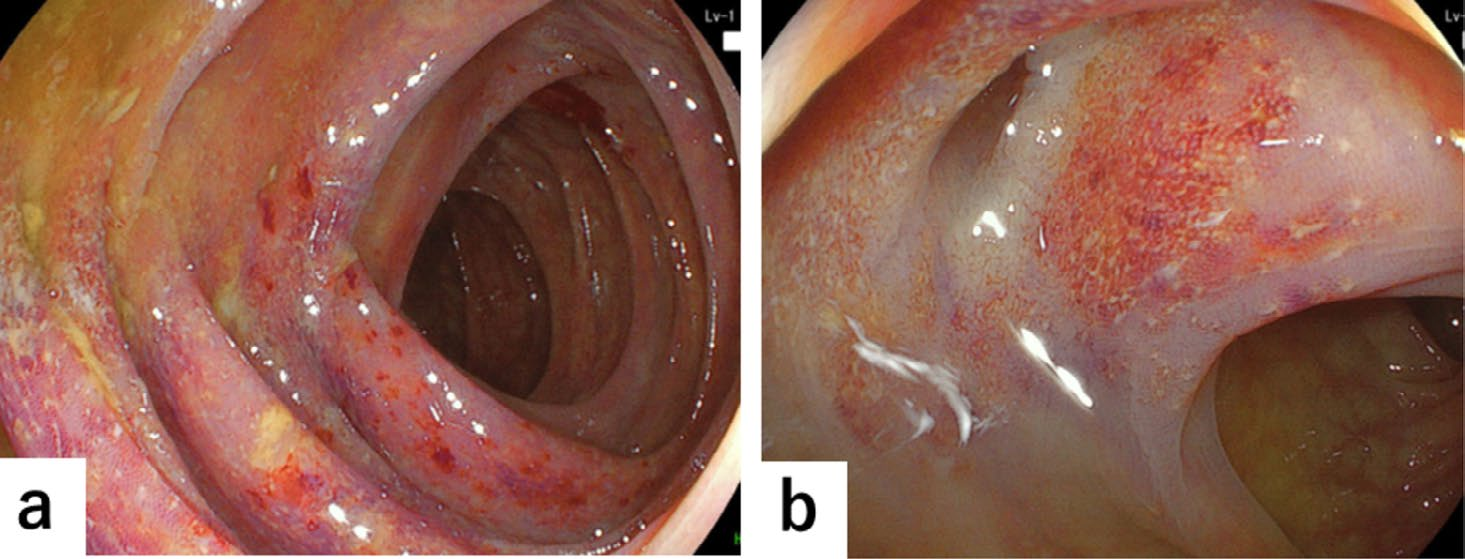
Colonoscopy images showing (a) diffuse fine granular mucosa with skip lesions throughout the colon, suggesting ulcerative colitis (UC) and (b) Multiple inflamed diverticula with surrounding mucosal inflammation, raising suspicion of concomitant diverticular disease.

Despite a 6‐week trial with cernitin pollen extract and minocycline, the symptoms persisted. Further patient history taking revealed chronic hematochezia, necessitating a lower gastrointestinal endoscopy that showed fine granular mucosa with skip lesions throughout the colon, consistent with UC (Figure 2a). Multiple diverticula with surrounding inflammation also raised suspicion of concomitant diverticular disease (Figure 2b). Histopathological examination confirmed UC with crypt abscesses, basal plasmacytosis, and goblet cell depletion. Stool culture revealed negative results.

**FIGURE 3:**
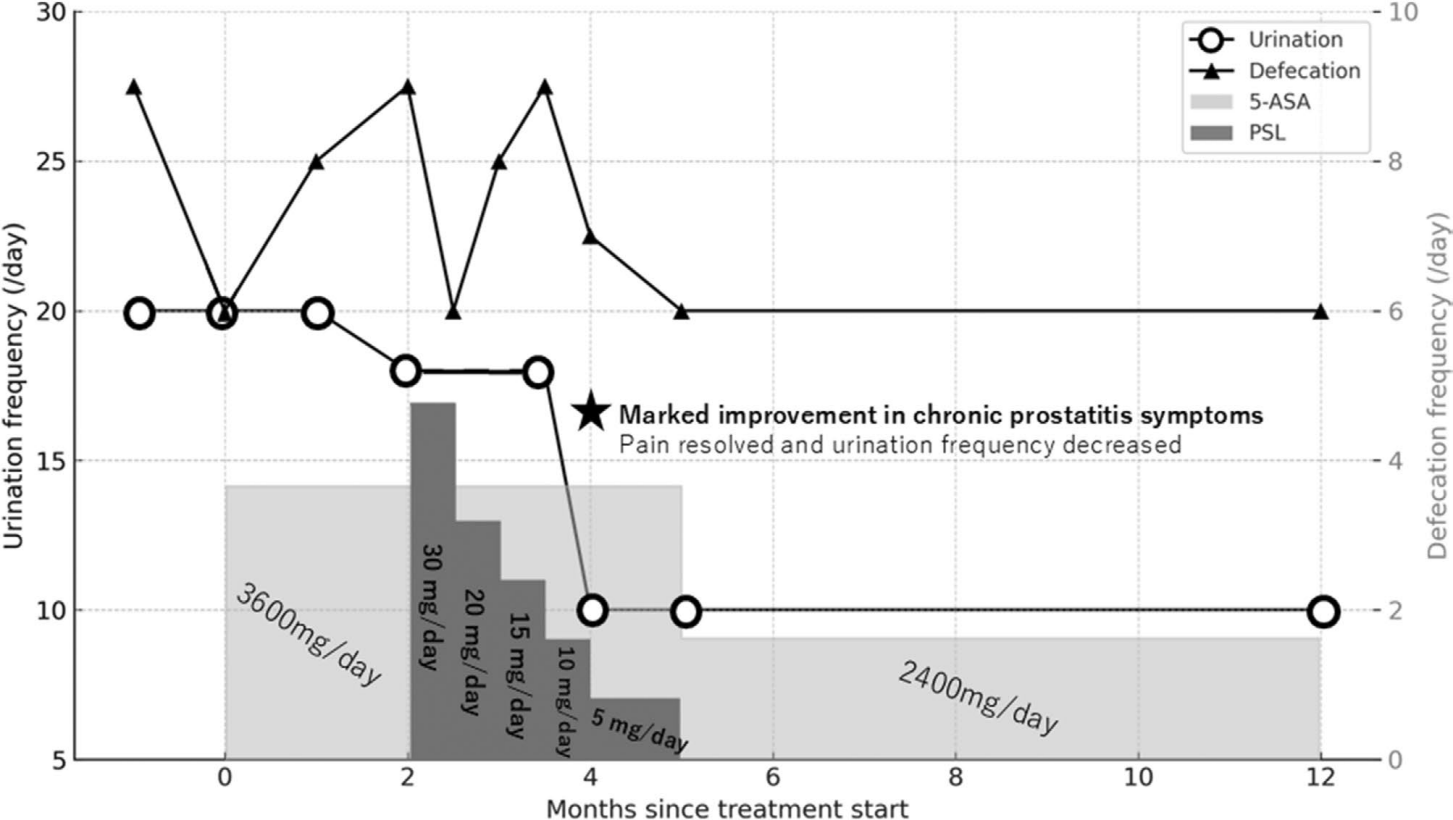
Clinical course of ulcerative colitis (UC) and urination symptom improvement. 5‐ASA, mesalazine; PSL, prednisolone.

Oral mesalazine treatment was initiated but failed to control the symptoms; thus, oral prednisolone was added (Figure 3). Approximately 1 month after starting prednisolone, the patient reported decreased urinary frequency and dysuria resolution by 3 months. One year later, a follow‐up colonoscopy confirmed remission. Although the hematochezia resolved, frequent defecation (up to eight times/day) persisted, suggesting comorbid irritable bowel syndrome (IBS).

The patient's urinary frequency improved concurrently, with intervals extending to 2 h. The NIH‐CPSI score decreased to 12 (pain: 0, voiding: 5, QoL: 7). All urological medications were discontinued; the patient's condition remained stable.

## 3 Discussion

This case highlights that chronic prostatitis might represent an EIM in a patient with UC. Although EIMs occur in approximately 27% of patients with UC, their involvement in the urological system remains poorly characterized [1]. Although mechanistic studies involving prostate biopsy would be ideal for verifying the pathophysiological link, such procedures were not performed in this case due to clinical improvement and the absence of malignancy. Typical EIMs in patients with UC include arthritis, dermatological lesions, ocular involvement, and hepatobiliary disorders. Although less common, urological involvement has also been reported [5]. EIM diagnosis is often clinical because many cases exhibit nonspecific features wherein establishing a direct causal relationship with UC could be challenging. This is a common limitation in cases of EIMs such as arthritis or ocular involvement, wherein histological confirmation of UC‐related inflammation is not typically feasible.

Several pathophysiological mechanisms may be linked to UC and chronic prostatitis. Both conditions involve upregulation of pro‐inflammatory cytokines, such as tumor necrosis factor (TNF‐)α, interleukin‐ (IL‐)1β, and IL‐6 [6, 7]. These mediators contribute to mucosal barrier dysfunction and immune hyperactivation. Moreover, the prostate's anatomical proximity to the rectum raises the possibility of direct spread of inflammation in patients with active UC. Whether prostatic inflammation could reflect contiguous spread from the rectum or systemic immune‐mediated mechanisms remains uncertain. Both scenarios are plausible, given the anatomical proximity and shared cytokine profiles. In older male populations with IBD, serum PSA levels are reportedly higher than in individuals without IBD, suggesting that local or systemic inflammatory responses associated with IBD influence the prostate [8].

Emerging literature has also implicated gut dysbiosis in both diseases. Alterations in the intestinal microbiota perpetuate mucosal inflammation in patients with UC and might also affect the prostate via the hypothesized “gut–prostate axis” [9, 10].

As demonstrated in this case, the coexistence of UC and chronic prostatitis might involve multiple pathophysiological mechanisms, including (1) shared pro‐inflammatory cytokine pathways, (2) contiguous spread of inflammation to adjacent organs, and (3) gut microbiota dysbiosis. These mechanisms suggest that chronic prostatitis, an EIM of UC, might be more common than currently recognized. In our case, symptom improvement following UC treatment supported the hypothesis that chronic prostatitis might sometimes reflect an EIM. Furthermore, urinary symptom improvement occurred only after initiating prednisolone, whereas mesalazine alone had little effect. This suggests that systemic corticosteroids might have played a more pivotal role. Prednisolone downregulates key pro‐inflammatory cytokines such as TNF‐α, IL‐1β, and IL‐6, which are implicated in the pathogenesis of both UC and chronic prostatitis.

Epidemiological data have shown increased LUTS in patients with IBD. One study reported a 26.8% prevalence of chronic prostatitis‐like symptoms in men with IBD; however, most were undiagnosed [11]. Case reports have shown chronic prostatitis resolution following rectal corticosteroid therapy for UC, suggesting a potential link [12].

In our case, coexisting diverticular disease was suspected. Diverticular disease is a clinical entity distinct from UC and characterized by chronic inflammation resembling that seen in UC, occurring in the mucosa between diverticula [13]. An association between diverticular disease and elevated TNF‐α has been shown, implying that TNF‐α–mediated mechanisms contributed to the pathophysiology observed in this patient [14].

Furthermore, despite achieving clinical remission, the patient continued to experience frequent bowel movements, raising the suspicion of comorbid IBS. A potential association between IBS and chronic prostatitis has also been reported; IBS might have contributed to the patient's persistently elevated NIH‐CPSI scores [15]. Conversely, prostatic inflammation might have extended to the rectum, thereby influencing bowel movement frequency. Further investigation is warranted to elucidate the complex interplay among UC, IBS, and chronic prostatitis.

## 4 Conclusions

This case highlights the importance of considering UC and other IBDs in the differential diagnosis of refractory chronic prostatitis, particularly when accompanied by gastrointestinal symptoms. The early recognition of this association might lead to more effective and targeted treatment strategies.

## Ethics Statement

This case report was conducted in accordance with the ethical standards of the Declaration of Helsinki. Ethics approval was not required for this single‐patient case report, as no experimental intervention was performed.

## Consent

Informed consent was obtained from the patient for the publication of this case report and the accompanying images.

## Conflicts of Interest

The authors declare no conflicts of interest.

## Data Availability

All data supporting the findings of this study are contained within the article. No additional datasets were generated or analyzed during the current study.
